# The U. S. Inspector in Our Large Stock-Yards1Read at the Thirty-eighth Annual Convention of the American Veterinary Medical Association, Atlantic City, N. J., September, 1901.

**Published:** 1901-12

**Authors:** S. G. Hendren

**Affiliations:** U. S. Inspector, Bureau of Animal Industry, New York


### THE U. S. INSPECTOR IN OUR LARGE STOCK-YARDS.[1]

By S. G. Hendren, V.M.D.,
U. S. INSPECTOR, BUREAU OF ANIMAL INDUSTRY, NEW YORK.

In offering a paper of this nature to the Society, I am aware that it is somewhat in the nature of an innovation, because it must of necessity be of a much less scientific type than most papers usually offered at such meetings. Yet I feel that its importance is manifest, since it is purely a practical paper, and one that can be applied by the practitioner as well as the inspector, for no veterinarian—no matter in what capacity he may be serving—can be too familiar with the nature of live-stock and the methods of handling them. When a practitioner I often wondered exactly what methods were employed by the United States Inspectors of the Bureau of Animal Industry in the large abattoirs and great stock-yards of the West in handling the enormous number of animals that were being continually presented to them for inspection. This always remained a mystery to me until I entered the service and learned it by actual contact. Being an Eastern man myself, I know there are a few busy practitioners in the East, and some who are thinking of entering the service, who would like to know something about how this work is carried on. I was thus induced to write a brief description of the duties and troubles of a United States Veterinary Inspector in a large stock-yard. It is simply one little corner of the vast work the Bureau of Animal Industry is doing, but it is sufficient to show what an inspector is supposed to be and do.

The rapid methods employed by the stock-yard companies are necessary in order to dispose of the great number of animals they receive, and these conditions require that the inspector be an active man and a quick observer. He must be a man who has had practical experience among live-stock, and plenty of it, and a liberal supply of ordinary common-sense and good judgment will be of great value to him in this work. He must have eyes that see, ears that hear, hands that feel, brains that think, and last, but not least, legs that walk. These requirements are very necessary for a veterinary inspector, for he must perform his duties in such a manner as not to delay the great volume of business that is waiting to be done. He will also find many pitfalls prepared for him,

[1] Read at the Thirty-eighth Annual Convention of the American Veterinary Medical Association, Atlantic City, N. J., September, 1901.

and he must be equal to the occasion when he meets them, otherwise he will find himself in many embarrassing and unpleasant positions. The inspector who does not like work very well had better not aspire to stock-yard honors, for he will never receive them.

The duties of the inspector in this line of work require that he be at his post at all times. It will not suffice for him to be there during the busy hours, or, worse yet, only on the busy days, but he must be there every day, and all the day, early and late, rain or shine. He must be at the chute to welcome the early arrivals, and at the scale to say farewell to the stragglers. The morning is the busy time at the stock-yard. You will find very little excitement after 2 P.M. during the busy period. The inspector is a very busy man. You may find him in a pen inspecting a bunch of suspicious animals. They may be "cholera hogs," "scabby sheep," or "actinomycotic cattle." You may find him at the scale passing over animals that have been sold, or you may find him in the alley looking for the fellow who is driving a "bunch of crooked stuff" around, trying to elude the government man.

Other duties that you will find him busily engaged in are the quarantining and dipping of sheep suffering from scab; the inspection and tagging of export animals; the quarantining of Southern tick-bearing cattle, so that they will do no harm in the way of spreading Texas fever, and the cleaning and disinfecting of all cars and pens that have contained any animals suffering from any disease of an infectious nature. These are some of the duties, and they require many steps in a day. The general diseases and conditions usually found, and for which animals are rejected, are advanced pregnancy, parturiency, abortion, emaciation, debility, exhaustion, excessive injuries, tumors, cancerous growths, and the commonly found acute diseases.

The specific diseases most commonly met with are: in hogs—hog cholera and swine-plague; sheep—sheep scab and foot-rot; cattle—actinomycosis and tuberculosis. These decisions and rejections are not made, however, without many trials and tribulations to the inspector, for many are the objections forthcoming from the owner and commission man if they think they have any chance of persuading you to change your mind. An inspector must be sure of his ground before he acts. He must be careful and not reject any cattle for actinomycosis which have nothing the matter with them but a neat supply of corn or hay packed away between their cheeks and molar teeth, and which may resemble very much a morbid growth. He must also beware of the car-load of pigs which have shipped very badly, and are crampy and "slow," commonly called "dumpy." They resemble very much cholera hogs, but recover very nicely when once penned, fed, and watered. But these matters must not deter an inspector from acting when he meets a real diseased condition. He must then make his diagnosis and stick to it. If it is actinomycosis, you will have the owner and commission man telling you all about how that lump was produced—by a "bad tooth," "wire-cut," "horse kick," "snake-bite," "scratched on the manger," "struck with a stone," etc.; or if it is confined to the region of the throat, they may explain that it is a bunch of "tongue fat" or "trough eggs." They will tell you anything if they think you will believe them and pass their animals. And then we have the man with the cholera hog which has just been laid on in the car, but has nothing else the matter with it; and the honest farmer with a cow that was not bred and has no calf in her, when the inspector can hardly lift it in his examination, but it is remarkable how the conscience of this same man will smite him about selling such a cow to the butcher when springers are scarce and bringing high prices, for then a government tag means quicker sales and more money to him.

The "scabby sheep," that the dogs have been tearing the wool out of, and the "bob calf," that is just eight weeks old by the almanac, are also to be found. This thing has become so popular that even the ministry has taken it up; and now we find the country parson with a bunch of pregnant sows, and yet, according to his story, he never had them off his place and never had a boar on the farm. These are a few of the trials the inspector must meet, but they are by no means all. He also has the scalper to deal with, and he is always ready with his little tale about where he just saw some "lumpy-jaw cattle," "scabby sheep, or "cholera hogs," according to which story he needs and wants to buy that day.

The feeder looking for stockers has his mind's eye so distorted by his future prospects that he can see nothing but "piggy sows," "calfy cows," "lamby ewes," and "bob calves" all around him. This is all human nature, and has become nothing but a cold business proposition to these men. They are each working for their own welfare. We cannot expect a man in the scalping business to understand why we refuse to reject a certain lot of animals when he knows that if he can get tags in their ears he can buy them for one-half their value. Neither can we expect the commission man to feel a calf in a cow within a few days of birth when the cow is a good beefer and worth five cents a pound. It is human nature all through, and the inspector must be constantly on the alert or he will get into a hornets' nest every hour in the day. He must not only be familiar with the handling of stock, and know their habits and ways, but he must also be familiar with the handling of stock-men and know their habits and ways. To permit one man to tell you a funny story while another drives some suspicious animals on the scale will subject an inspector to much ridicule.

When government inspection was first introduced at the stock-yards it did not meet with a very warm reception. It was a very hard matter for commission men, stock-owners, and buyers to understand why the requirements of the United States inspection should be so strict. Their State and city did not enforce any such rule, and they could not see why the government should; but 99 per cent. of those men to-day admire the work being done by the government, and commend the inspector of the United States Bureau of Animal Industry for the manner in which he performs his duty.

The government inspection is no farce; it is inspection that inspects. It is carried on by an organized force in a systematic manner, according to scientific principles. It accomplishes what it sets out to do, and that is the suppression and eradication of contagious and infectious diseases among the live-stock of our country and the promotion of health among them.

---

STRAMONIUM FOR HORSE FLIES. The following translation from *Le Bulletin Général de Thérapeutique* is taken from the *Pharmaceutical Journal:* According to the *Chasseur Illustré*, a decoction of one part of stramonium leaves to three parts of water, boiled for twenty minutes, and applied, when cool, to the face, about the ears, inside the legs, about the belly and croup, is sufficient to keep a horse free from its tormentors during a whole day. Stramonium is said to be much more efficacious when thus used than tobacco. The plant referred to is probably the common "thorn-apple" or "stinkblaar" of Cape Colony—Datura Stramonium, Linn.—Editor *Agricultural Journal*, October 24, 1901.

